# Developing a Metabolic-Associated Prognostic Index for Risk Stratification and Therapeutic Guidance in Stage I Lung Adenocarcinoma via Multiomics Analysis

**DOI:** 10.1200/PO-25-00897

**Published:** 2026-03-02

**Authors:** Pengcheng Liu, Zhongxu Chen, Hui Hong, Yihua Sun

**Affiliations:** ^1^ Department of Thoracic Surgery and State Key Laboratory of Genetic Engineering, Fudan University Shanghai Cancer Center, Shanghai, China; ^2^ Institute of Thoracic Oncology, Fudan University, Shanghai, China; ^3^ Department of Oncology, Shanghai Medical College, Shanghai, China

## Abstract

**PURPOSE:**

Early-stage lung adenocarcinoma (LUAD) exhibits substantial clinical heterogeneity that is not fully explained by TNM staging, highlighting the need for biology-driven prognostic tools. Although metabolic reprogramming is an established cancer hallmark, its systematic prognostic significance in stage I LUAD remains unexplored.

**MATERIALS AND METHODS:**

We analyzed stage I LUAD samples from The Cancer Genome Atlas, Gene Expression Omnibus, and European Genome-phenome Archive databases. Weighted gene coexpression network analysis and differential expression analysis were conducted to identify metabolic genes associated with LUAD malignancy and prognosis. A metabolic-associated prognostic index (MAPI) was subsequently developed using machine-learning combinations. The performance of MAPI was evaluated from multiple biologic perspectives and at the single-cell level. Key gene functions were experimentally verified in vitro.

**RESULTS:**

MAPI robustly stratified patients into high- and low-risk groups with significantly divergent survival outcomes, outperformed conventional clinicopathologic features and previously published signatures, and emerged as an independent prognostic factor across all validation cohorts. The high-risk group was characterized by enhanced cancer stemness, genetic heterogeneity, metabolic reprogramming, immune exclusion, and reduced responsiveness to immunotherapy. We also pinpointed three potential therapeutic agents (paclitaxel, bortezomib, and vincristine) for high-risk patients. The single-cell RNA sequencing further validated the association between MAPI and malignant progression. Functional analyses demonstrated that knockdown of *DEGS1* or *PLOD1* significantly suppressed LUAD cell proliferation and migration.

**CONCLUSION:**

Our results establish MAPI as a biologically interpretable and clinically applicable tool for risk stratification and precision treatment decision making in patients with stage I LUAD.

## INTRODUCTION

Lung cancer is the foremost cause of cancer-related mortality worldwide,^1^ with lung adenocarcinoma (LUAD) constituting its predominant histologic subtype.^2^ Although low-dose computed tomography screening has enhanced the identification of stage I LUAD,^3^ its long-term prognosis remains unsatisfactory, with 5-year overall survival (OS) rates of 67%-90% and a recurrence risk of approximately 17.9%.^4–6^ Therefore, characterizing the biologic features of early-stage LUAD and identifying high-risk patients who may benefit from specific therapies are essential to maximize the advantages of early detection and improve survival.

**CONTEXT**

**Key Objective**
Patient survival in stage I lung adenocarcinoma (LUAD) remains unsatisfactory despite advances in early detection. This study aimed to develop a metabolic-associated prognostic index (MAPI) to identify high-risk patients and support treatment decision making in stage I LUAD.
**Knowledge Generated**
We developed an MAPI using machine-learning combinations and validated it across multiple independent cohorts. MAPI stratified patients with stage I LUAD into high-risk and low-risk groups with significant different survival, molecular characteristics, immune phenotypes, and therapeutic vulnerabilities.
**Relevance**
MAPI allows for the identification of patients with high-risk stage I LUAD who may benefit from specific therapeutic agents, facilitating personalized treatment strategies to improve survival.

Malignant cells universally reprogram their metabolism in response to intrinsic alterations and extrinsic microenvironmental stress.^7^ Previous studies have highlighted the importance of metabolic reprogramming in LUAD, even at its earliest stage.^8,9^ Furthermore, distinct metabolic subtypes with unique clinical characteristics have been identified within LUAD.^8^ These findings suggest metabolic reprogramming as a valuable foundation for prognostic modeling to refine risk stratification in early-stage LUAD. However, despite increasing interests in metabolism-related phenotypic indices in LUAD,^10,11^ metabolic prognostic models specifically designed for stage I LUAD remain lacking.

To address this gap, we established the metabolic-associated prognostic index (MAPI). The overall workflow is shown in the Data Supplement (Fig S1). Using weighted gene coexpression network analysis (WGCNA) and machine-learning algorithm combinations, we identified crucial metabolic genes and constructed optimal prognostic model for stage I LUAD. MAPI could divide patients with stage I LUAD into two groups with distinct survival, molecular characteristics, genetic alterations, immune microenvironments, and therapeutic vulnerabilities. The robustness of MAPI was validated in different data sets and at single-cell resolution. Functionally, we found that targeting *DEGS1* and *PLOD1*, core components of MAPI, could significantly inhibit the growth and migration of LUAD cells. Collectively, our research supports the risk stratification of stage I LUAD and the implementation of personalized treatment, with the potential to improve patient prognosis.

## MATERIALS AND METHODS

### Data Acquisition

We collected 3,663 metabolic genes from KEGG, REACTOME, and Human-GEM databases.

A total of 550 patients with stage I LUAD from four independent cohorts were included. Patients with a follow-up time of less than 3 months were excluded. The Cancer Genome Atlas (TCGA) cohort (N = 234) served as training cohort, whereas GSE30219 (N = 78),^13^ GSE31210 (N = 168),^14^ and Fudan University Shanghai Cancer Center (FUSCC) (EGAS00001004006; N = 70)^15^ cohorts were used as validation cohorts. The maximum expression value was retained for genes detected by multiple probes. Metacohort was created by merging the four cohorts, and batch effects were corrected using R package sva (3.48.0). Baseline characteristics of the four cohorts are summarized in the Data Supplement (Table S1).

For the validation of predicted immunotherapy response, 25 patients receiving adoptive T-cell therapy from GSE100797 cohort^16^ were included. For the validation of MAPI at single-cell resolution, nine patients with early-stage LUAD (three adenocarcinoma in situ [AIS], three minimally invasive adenocarcinoma [MIA], and three invasive adenocarcinoma [IAC]) from GSE189357 cohort^17^ were included.

### Machine-Learning Algorithms for the Construction of the Metabolic-Associated Prognostic Index

R package Mime1 (v.0.0.0.9000) was used to construct MAPI. Briefly, we applied 10 classical machine-learning algorithms—Stepwise Cox (StepCox), Ridge, Supervised Principal Components, Elastic Net, CoxBoost, Random Survival Forest (RSF), Lasso, Partial Least Squares Cox Regression, Survival Support Vector Machine algorithms, and Generalized Boosted Regression Model—as well as their combinations (Data Supplement, Table S2), to build prognostic models for disease-free survival (DFS). Among these, StepCox, CoxBoost, RSF, and Lasso possess intrinsic capabilities in variable selection, facilitating their combination with other algorithms. The training data were the expression profile of patients with stage I LUAD in the TCGA cohort, and the features used were derived from the intersection of metabolic genes linked to early-stage LUAD malignancy and differentially expressed genes (DEGs) between early-stage LUAD tumors and normal tissues. Then, the concordance index (C-index) of each model was calculated in both training and validation cohorts using R package survival (v3.5-5) with DFS as the reference, and the most robust model was selected as the final MAPI model.

## RESULTS

### Identification of Coexpressed Metabolic Modules Associated With Early-Stage LUAD Malignancy

To build MAPI for early-stage LUAD, we collected patients with stage I LUAD in the TCGA training cohort and three validation cohorts. On the basis of the metabolic genes from KEGG, REACTOME, and Human-GEM databases (Data Supplement, Fig S2A), we performed WGCNA in the TCGA cohort. Using a soft threshold power of 5, we identified 17 distinct modules (Data Supplement, Figs S2B and 2C). Module-trait correlations across sex, age, relapse, death, and stage revealed nine modules significantly associated with early-stage LUAD malignancy (Data Supplement, Fig S2D): Modules 6 and 12 correlated with relapse; Module 7 with death; Modules 1, 4, 6, and 17 with stage; and Modules 13, 15, and 16 with all three traits.

Across these modules, 670 metabolic genes were linked to early-stage LUAD malignancy. We next investigated the function of genes within the nine modules (Data Supplement, Fig S2E) and found that Modules 1 and 13 were enriched for cell respiration, Module 4 for fatty acid metabolism and nucleotide metabolism, Module 6 for toxin response, Module 7 for bioactive lipid mediator metabolism, Module 12 for glycerophospholipid metabolism, Module 15 for serine metabolism, Module 16 for phospholipid metabolism, and Module 17 for protein and RNA homeostasis.

Furthermore, we identified 25,300 DEGs between early-stage LUAD tumors and normal tissues, including 2,629 metabolic genes (Data Supplement, Fig S2F). Intersecting the metabolic gene set, trait-associated gene set, and DEGs yielded 548 core metabolic genes for MAPI construction (Data Supplement, Fig S2G and Table S3). These genes were enriched in the cofactor, carbon and amino acid metabolism (Data Supplement, Fig S2H).

### Construction and Validation of the Metabolic-Associated Prognostic Index

**FIG 1.**
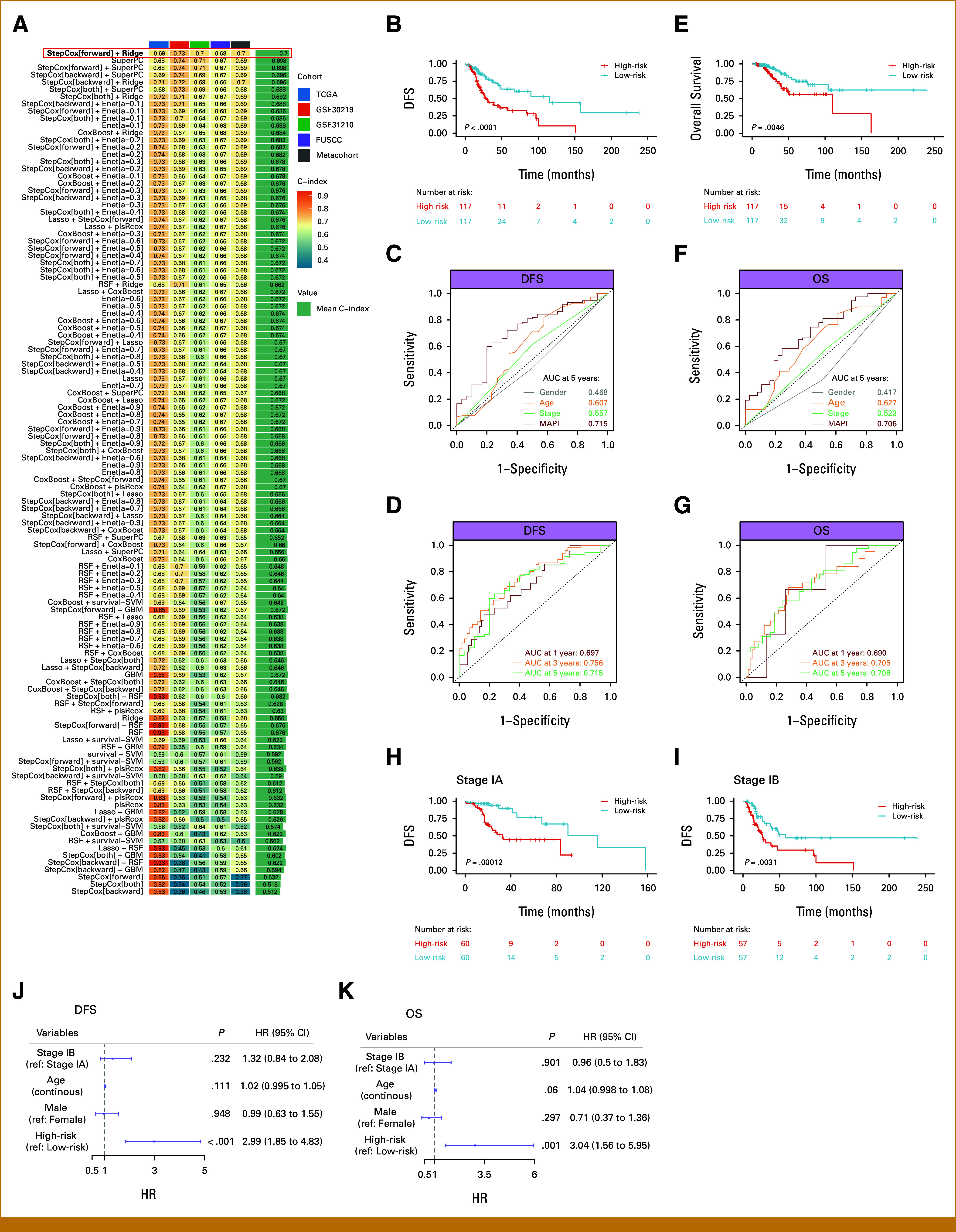
Construction and validation of the metabolic-associated prognostic index. (A) C-index of each model across the four cohorts. The combination of StepCox[forward] and Ridge was chosen as the final MAPI model. (B, E) Kaplan-Meier curves for DFS (B) and OS (E) between the MAPI groups in the TCGA cohort. (C, F) ROC curves comparing MAPI with clinicopathologic variables for predicting 5-year DFS (C) and OS (F). (D, G) Time-dependent ROC curves for 1-, 3-, and 5-year (D) DFS and (G) OS prediction by MAPI. (H, I) Kaplan-Meier curves for DFS by MAPI stratification in stage IA (H) and stage IB (I) patients. (J, K) Multivariable Cox analysis for (J) DFS and (K) OS. DFS, disease-free survival; HR, hazard ratio; MAPI, metabolic-associated prognostic index; OS, overall survival; TCGA, The Cancer Genome Atlas.

As described in methods, we used machine-learning algorithm combinations to develop models for DFS using the TCGA training cohort and then calculated C-index for each model in all cohorts. Ultimately, the combination of StepCox[forward] and Ridge was chosen as the final MAPI model as it achieved the highest C-index (Fig 1A; Data Supplement, Table S4).

Patients with early-stage LUAD in the TCGA cohort were divided into high- and low-risk groups according to the median value of calculated MAPI scores. As expected, high-risk patients exhibited significantly shorter DFS (Fig 1B). Receiver operating characteristic (ROC) curves showed that MAPI outperformed clinicopathologic variables in DFS prediction (Fig 1C). Time-dependent ROC curves revealed AUC values of 0.697, 0.756, and 0.715 for 1-, 3-, and 5-year DFS, respectively (Fig 1D), confirming robust prognostic power. Strikingly, MAPI also effectively stratified OS, with superior survival in the low-risk group (Fig 1E). Similarly, AUC values for 1-, 3-, and 5-year OS were 0.690, 0.705, and 0.706, respectively, exceeding clinicopathologic parameters (Figs 1F and 1G).

Subgroup analysis demonstrated consistently better DFS in the low-risk group across tumor stages (Figs 1H and 1I). Critically, multivariable Cox analyses validated MAPI as an independent predictor for both DFS and OS (Figs 1J and 1K; Data Supplement, Figs S3A and S3B). Collectively, these results indicate that MAPI provides strong prognostic value for patients with early-stage LUAD.

### Generalizability of MAPI in Independent Validation Cohorts

**FIG 2.**
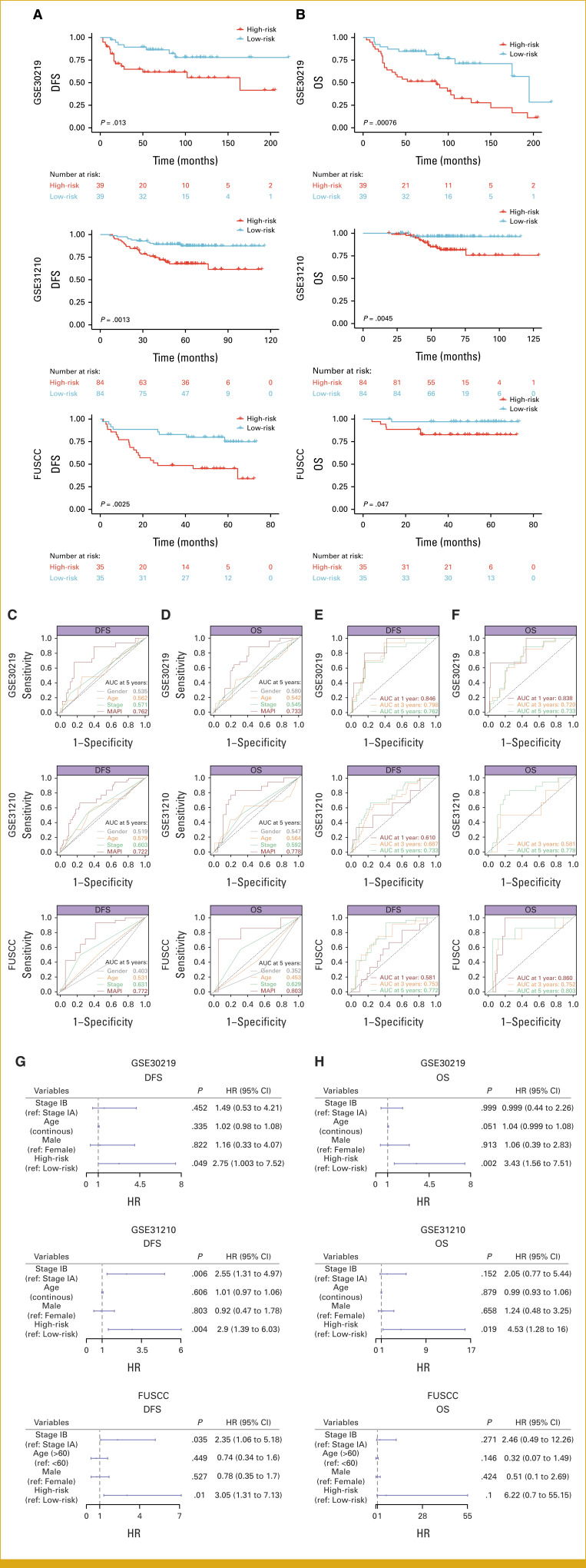
Generalizability of MAPI in independent validation cohorts. (A, B) Kaplan-Meier curves for DFS (A) and OS (B) between the MAPI groups in the GSE30219 (top), GSE31210 (middle), and FUSCC cohorts (bottom). (C, D) ROC curves comparing MAPI with clinicopathologic variables for predicting 5-year (C) DFS and (D) OS across the three cohorts. (E, F) Time-dependent ROC curves for 1-, 3-, and 5-year (E) DFS and (F) OS prediction by MAPI across the three cohorts. (G, H) Multivariable Cox analysis for (G) DFS and (H) OS across the three cohorts. DFS, disease-free survival; FUSCC, Fudan University Shanghai Cancer Center; HR, hazard ratio; MAPI, metabolic-associated prognostic index; OS, overall survival.

We next evaluated the generalizability of MAPI in validation cohorts using the same median-based cutoff. Survival analysis revealed significantly worse DFS and OS in the high-risk group across all three cohorts (Figs 2A and 2B). ROC curves demonstrated excellent performance of MAPI over clinicopathologic features, with AUC values for 1-, 3-, and 5-year DFS of 0.846, 0.798, 0.762 in the GSE30219 cohort; 0.610, 0.687, and 0.733 in the GSE31210 cohort; and 0.581, 0.753, and 0.772 in the FUSCC cohort (Figs 2C and 2E). Similarly, AUC values for 1-, 3-, and 5-year OS were 0.838, 0.720, and 0.733 in the GSE30219 cohort and 0.860, 0.752, and 0.803 in the FUSCC cohort (Figs 2D and 2F). In the GSE31210 cohort, where no deaths occurred within the first year, AUC values for 3- and 5-year OS were 0.581 and 0.778, respectively (Figs 2D and 2F). Notably, multivariable Cox analyses corroborated MAPI as an independent prognostic factor for both DFS and OS across the three cohorts (Figs 2G and 2H; Data Supplement, Figs S4A, and S4B).

Furthermore, we compared C-index of MAPI model with that of previously established prognostic models (Data Supplement, Table S5) and found that the performance of MAPI ranked among the top in all cohorts (Data Supplement, Fig S4C).

### Distinct Molecular Characteristics and Genetic Alterations Between MAPI Risk Groups

**FIG 3.**
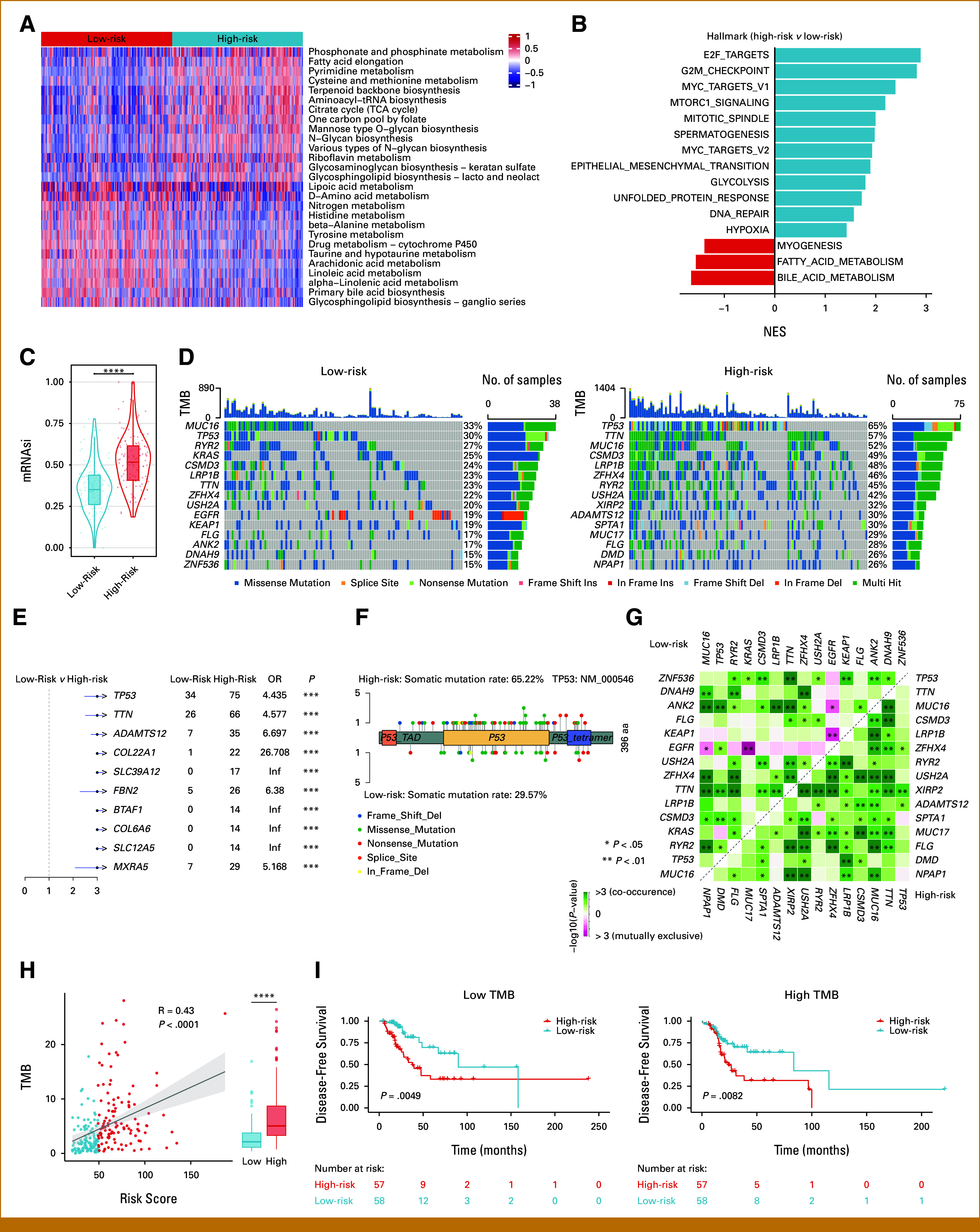
Molecular characteristics and genetic alterations across MAPI groups. (A) Heat map showing the differentially enriched KEGG metabolic pathways (|log_2_ FC| > .2 and an adjusted *P* value <.05) between MAPI groups. (B) Gene set enrichment analysis (H: hallmark gene sets) of altered pathways (adjusted *P* value <.05) between MAPI groups. (C) Comparison of mRNAsi between MAPI groups in the TCGA cohort. (D) Top 15 recurrently mutated genes in the two MAPI groups. (E) Top 10 differentially mutated genes between MAPI groups. (F) Distribution of *TP53* mutation sites in the two MAPI groups. (G) Landscape of co-occurring or mutually exclusive mutated gene pairs. (H) Scatter plot showing the correlation between TMB and MAPI risk score. (I) Kaplan-Meier curves for DFS by MAPI stratification in low- (left) and high-TMB (right) patients. *P < .05; **P < .01; ***P < .001; ****P < .0001. DFS, disease-free survival; MAPI, metabolic-associated prognostic index; TCGA, The Cancer Genome Atlas.

Given the outstanding prognostic efficacy of MAPI, we intended to elucidate the biologic mechanisms underlying the risk stratification. As metabolic reprogramming is a hallmark of cancer,^18^ we first quantified the KEGG metabolic pathway activities for each sample. Glycan metabolism was significantly increased in the high-risk group (Fig 3A), in line with its role in multiple cancer-related biologic processes, such as cell-matrix interactions and immune surveillance.^19^ Besides, metabolism of cofactors and vitamins, including lipoic acid metabolism, was also elevated in the high-risk group (Fig 3A). Conversely, lipid programs, as well as primary bile acid biosynthesis, were enriched in the low-risk group (Fig 3A). Gene set enrichment analysis of hallmark gene sets similarly highlighted fatty acid metabolism and bile acid metabolism in the low-risk group (Fig 3B). Together, these results indicate a role for metabolic reprogramming in MAPI-defined risk divergence.

Beyond metabolism, the high-risk group exhibited upregulation of pathways related to cell proliferation, migration, and resistance to cell death (Fig 3B), all of which are associated with tumor stemness and linked to malignancy progression.^20^ Therefore, we adopted a widely recognized OCLR algorithm to assess tumor stemness. Interestingly, the high-risk group showed higher mRNAsi scores in all cohorts (Fig 3C; Data Supplement, Figs S5A-S5C), indicative of strengthened tumor stemness.

We next explored genetic alterations between MAPI groups. The top 15 recurrently mutated genes in the two groups varied (Fig 3D). Notably, the top 10 differentially mutated genes were all more frequent in the high-risk group (Fig 3E). *TP53*, a key tumor suppressor gene, displayed the largest disparity (Fig 3F), implying accelerated tumor progression and poor prognosis in the high-risk group.^21^ We also observed that the significantly co-occurring or mutually exclusive mutated gene pairs diverged between the two groups (Fig 3G).

Additionally, we quantified the mutation level in the genome and found that the high-risk group harbored a higher tumor mutation burden (TMB; Fig 3H). Also, TMB correlated positively with MAPI scores (Fig 3H). Subgroup analysis revealed that the high-risk group showed inferior DFS irrespective of TMB (Fig 3I). Therefore, these data underscore pronounced metabolic and genomic heterogeneity between MAPI groups, with the high-risk group exhibiting higher tumor stemness and TMB.

### Low-Risk Group Exhibits Enhanced Immune Infiltration and Potential Immunotherapy Benefit

**FIG 4.**
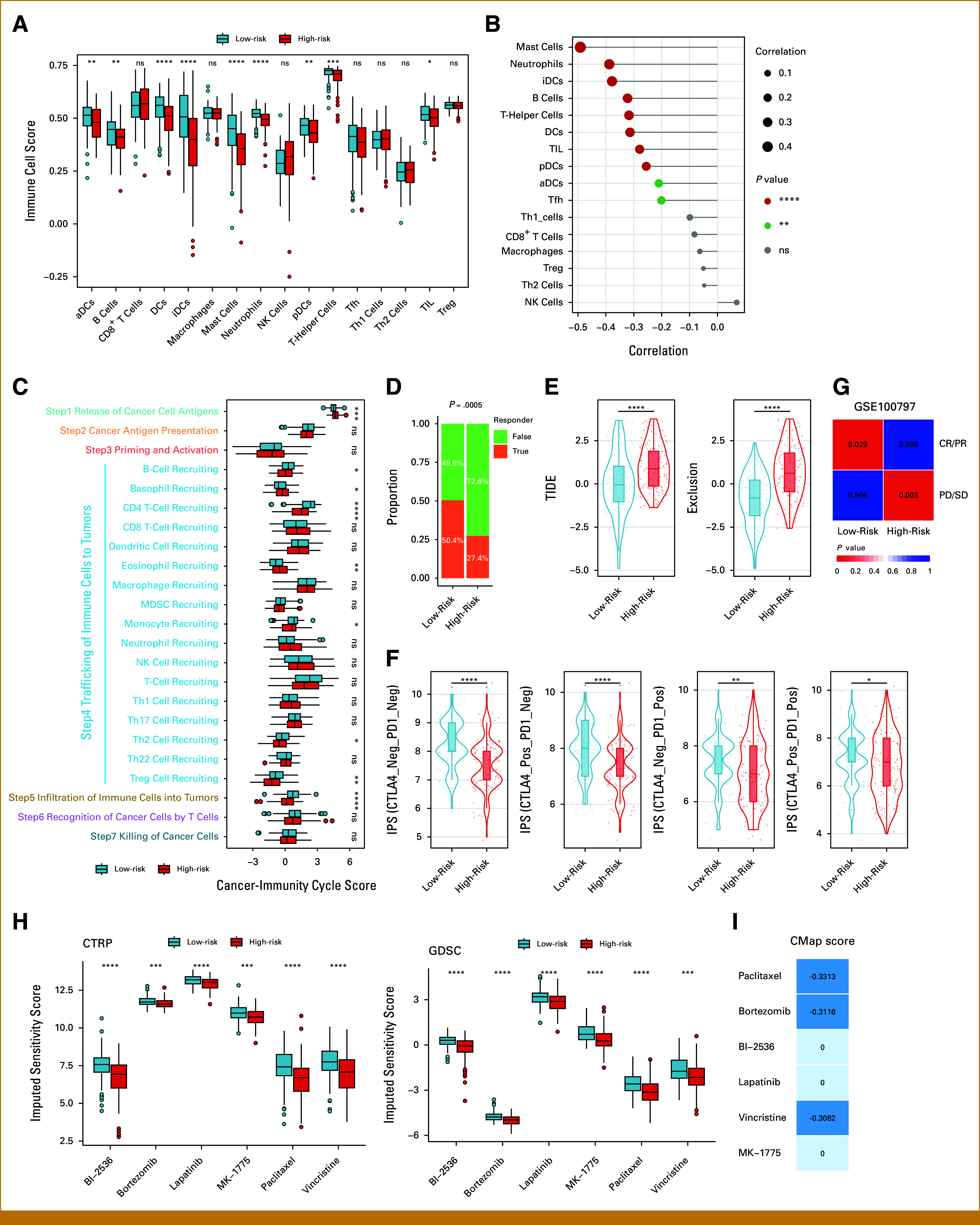
Immune landscape and therapeutic vulnerabilities of MAPI groups. (A) Box plot showing the abundance of tumor-infiltrating immune cells between MAPI groups in the TCGA cohort. (B) Lollipop plot showing the correlation between immune cell abundance and MAPI risk score. (C) Box plot showing the difference in cancer-immunity cycle activities between MAPI groups. (D) Composition proportion of immunotherapy response status between MAPI groups. (E) Comparisons of TIDE score (left) and exclusion score (right) between MAPI groups. (F) Comparisons of the four IPS scores between MAPI groups. (G) SubMap analysis comparing MAPI groups with GSE100797 subgroups. (H) Box plot showing the imputed sensitivity of six drugs from CTRP (left) and GDSC (right) databases between MAPI groups in the TCGA cohort. (I) Identification of the most promising drugs for the high-risk group based on the CMap database. *P < .05; **P < .01; ***P < .001; ****P < .0001; ns = *P* ≥ .05. CTRP, cancer therapeutics response portal; GDSC, genomics of drug sensitivity in cancer; MAPI, metabolic-associated prognostic index; TCGA, The Cancer Genome Atlas.

The tumor microenvironment (TME) plays a pivotal role in influencing tumor malignancy and treatment response.^22^ Thus, we investigated the immune phenotypes of MAPI groups. We first assessed the abundance of immune cell types in the TME.^23^ We found that most immune subsets, including dendritic cells, granulocytes, and lymphocytes, had increased infiltration levels in the low-risk group (Fig 4A), whereas none of the immune cells were elevated in the high-risk group (Fig 4A). In addition, MAPI scores correlated negatively with immune infiltration (Fig 4B). Validation cohorts exhibited similar pattern (Data Supplement, Figs S6A-S6C).

The immune response to cancer follows a sequential, self-reinforcing process termed cancer-immunity cycle, which is linked to immunotherapy response.^24^ We estimated the cancer-immunity cycle score and found that various steps were upregulated in the low-risk group, such as trafficking and infiltration of immune cells to tumors (Fig 4C), consistent with the observed increase in immune cell abundance in the low-risk group (Figs 4A and 4B).

Given the immune activation characteristics observed in the low-risk group, we speculated that the low-risk group may have benefitted from immunotherapy. To test our hypothesis, we adopted TIDE,^25^ an algorithm modeling two primary mechanisms of tumor immune evasion, to predict immunotherapy response. The low-risk group had significantly higher response rates and lower TIDE scores and exclusion scores (Figs 4D and 4E), suggesting less T-cell exclusion and a favorable response to immunotherapy. Then, we applied IPS, a scoring scheme developed from a panel of immune-related genes,^26^ to predict the response to immunotherapy. All the four IPS scores were higher in the low-risk group (Fig 4F), implying potential immunotherapy benefit. We confirmed these findings in validation cohorts (Data Supplement, Figs S6D-S6L).

Moreover, we used SubMap algorithm to compare our identified MAPI groups with a previous cohort that received adoptive T-cell therapy.^16^ Patients in the low-risk group resembled complete or partial responders, whereas high-risk patients resembled nonresponders (Fig 4G). Collectively, these results characterize the low-risk group as immunologically activate and potentially more responsive to immunotherapy.

### Therapeutic Screening for High-Risk Groups

Due to the predicted limited immunotherapy response in the high-risk group, we explored potential therapeutic alternatives using genomics of drug sensitivity in cancer and cancer therapeutics response portal drug sensitivity databases. The imputed sensitivity scores of six compounds, BI-2536, bortezomib, lapatinib, MK-1775, paclitaxel, and vincristine, were lower in the high-risk group and negatively correlated with MAPI scores, and exhibited robust performance in validation cohorts (Fig 4H; Data Supplement, Figs S7A-S7D), implying a better response to these drugs in the high-risk group. In addition, paclitaxel, bortezomib, and vincristine had CMap scores <0, suggesting the potential to reverse the high-risk transcriptional program toward a normal-like state (Fig 4I). These three agents therefore emerge as promising candidates for patients with early-stage LUAD in the high-risk group.

### Validation of MAPI at the Single-Cell Resolution

**FIG 5.**
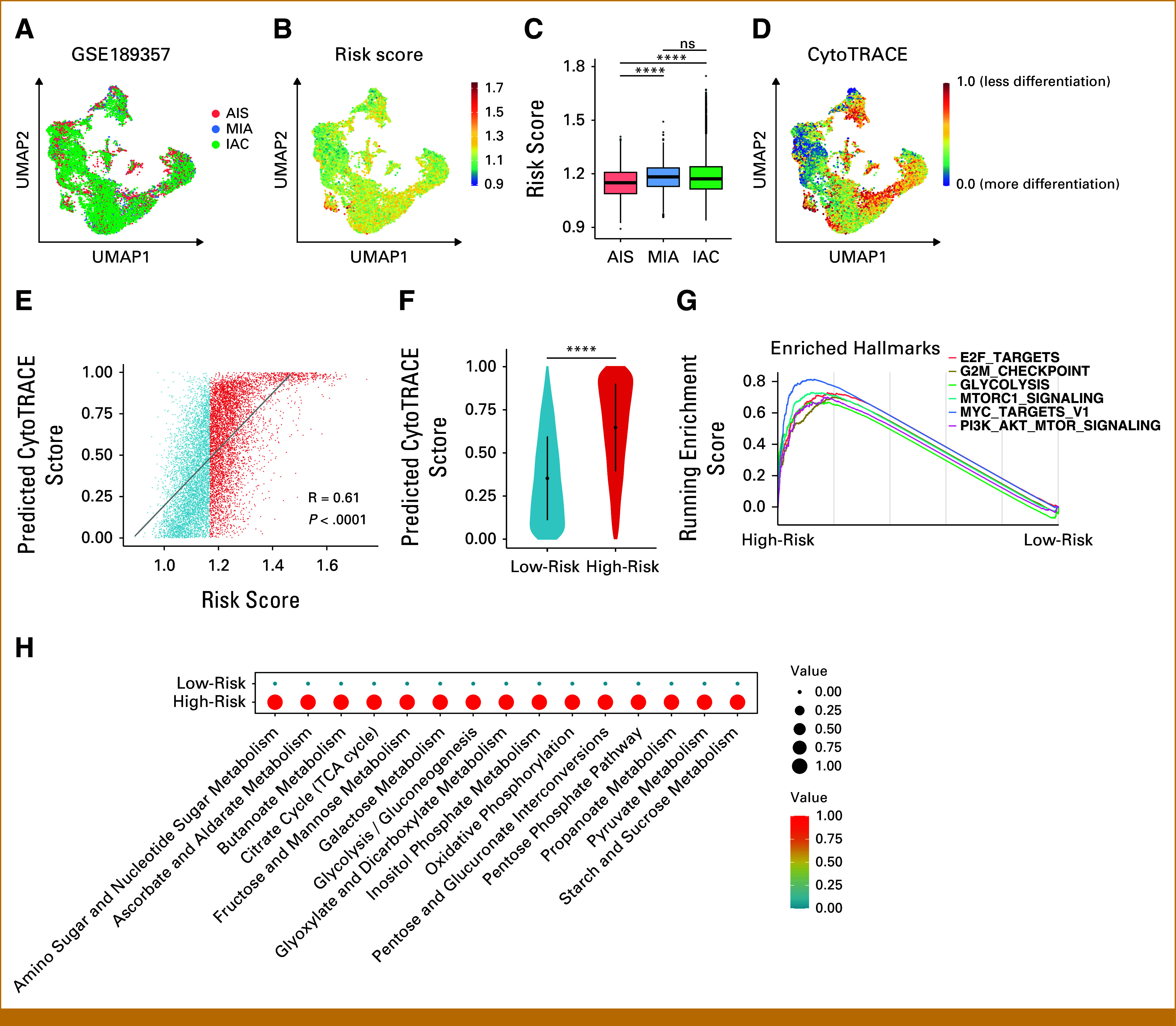
Validation of MAPI at the single-cell resolution. (A, B) UMAP plot of epithelial cells from the GSE189357 cohort colored by (A) pathologic subtypes or (B) MAPI risk score. (C) Comparisons of MAPI risk score across pathologic subtypes. (D) UMAP plot of epithelial cells from the GSE189357 cohort colored by CytoTRACE score. (E) Scatter plot showing the correlation between CytoTRACE score and MAPI risk score. (F) Comparison of the CytoTRACE score between MAPI groups. (G) Gene set enrichment analysis (H: hallmark gene sets) showed significantly enriched pathways (adjusted *P* value <.05) in the high-risk group. (H) Bubble plot showing the metabolic pathway activities between MAPI groups. ****P < .0001; ns = *P* ≥ .05. MAPI, metabolic-associated prognostic index; TCGA, The Cancer Genome Atlas; UMAP, Uniform manifold approximation and projection.

To evaluate MAPI at single-cell level, we analyzed a public scRNA-seq data set of early-stage LUAD, consisting of three patients with AIS, three patients with MIA, and three patients with IAC.^17^ Following epithelial cell extraction and integration, we calculated MAPI score per cell (Figs 5A and 5B). Notably, cells from MIA and IAC displayed higher MAPI scores (Fig 5C), suggesting a more aggressive phenotype. Next, we predicted cell differentiation state using CytoTRACE,^27^ with higher CytoTRACE scores indicating a more undifferentiated state and enhanced stemness. As anticipated, CytoTRACE scores were lower in low-risk cells and correlated positively with MAPI scores (Figs 5D-5F).

We next explored the biologic differences between cells in high-risk and low-risk groups. Concordant with Figure 3B, cells in the high-risk group upregulated pathways related to cell proliferation and energy metabolism (Fig 5G) and displayed globally enhanced metabolic activity (Fig 5H). These results substantiate the robustness and mechanistic plausibility of MAPI for prognostication in early-stage LUAD at single-cell resolution.

### Assessment of the Oncogenic Roles of *DEGS1* and *PLOD1*

**FIG 6.**
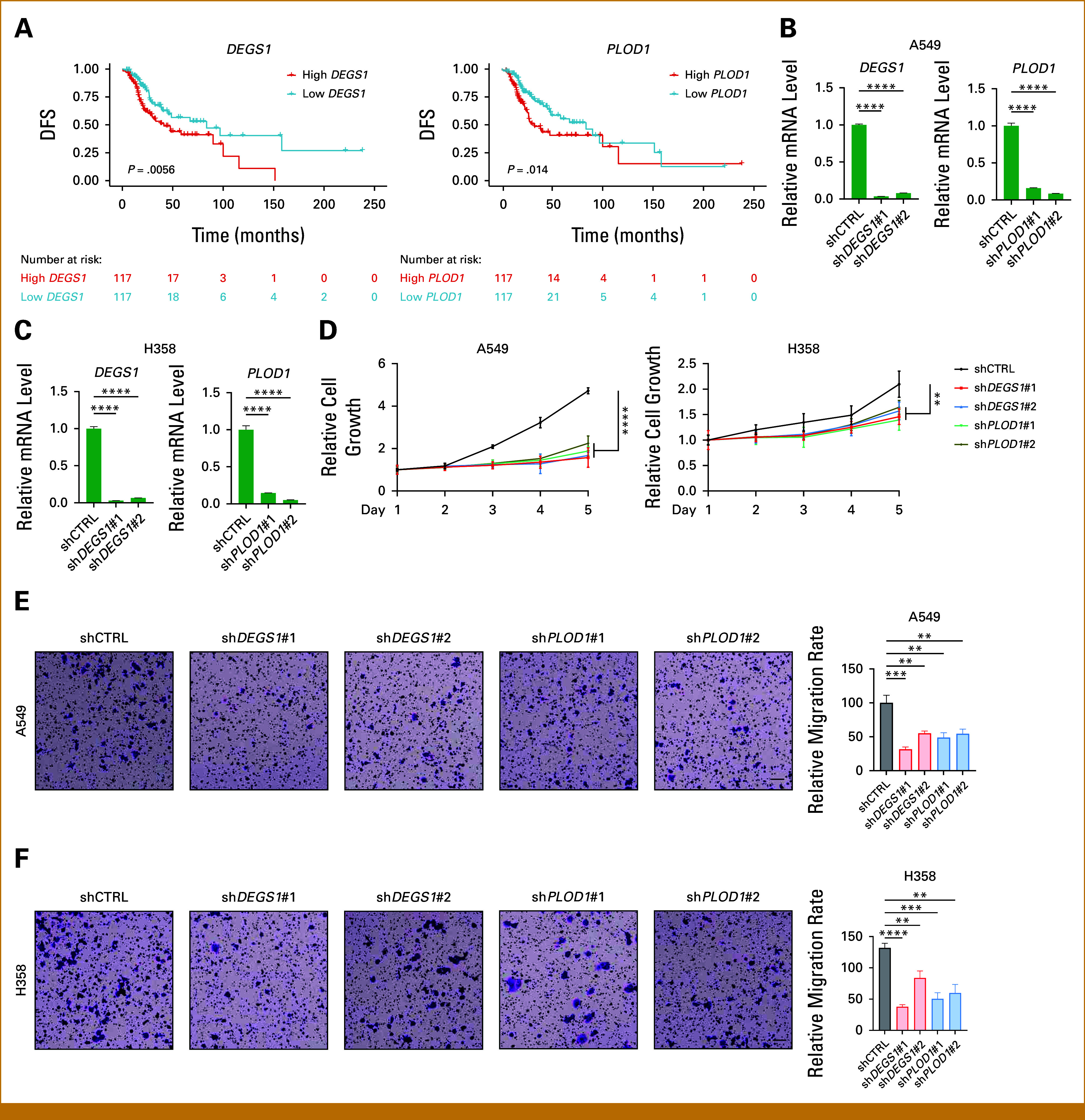
Assessment of the oncogenic roles of *DEGS1* and *PLOD1*. (A) Kaplan-Meier curves for DFS in the TCGA cohort stratified by the expression of *DEGS1* (left) and *PLOD1* (right). (B, C) The expression of *DEGS1* or *PLOD1* on (B) A549 and (C) H358 cells knocking down *DEGS1* or *PLOD1* (n = 3). Data represent mean ± SD. (D) Cell proliferation assays were performed on A549 (left) and H358 (right) cells transfected with shCTRL, sh*DEGS1*, or sh*PLOD1* (n = 5). Data represent mean ± SD. (E, F) Transwell migration assays were performed on (E) A549 and (F) H358 cells transfected with shCTRL, sh*DEGS1*, or sh*PLOD1* (n = 5). Representative images (scale bar: 100 μm) of the migrated cells (left) and statistical analyses (right) are shown. Data represent mean ± SD. **P < .01; ***P < .001; ****P < .0001. DFS, disease-free survival; MAPI, metabolic-associated prognostic index; SD, standard deviation; TCGA, The Cancer Genome Atlas.

Among all genes comprising MAPI, *DEGS1* and *PLOD1* contributed most to MAPI score and positively correlated with it, implying their potential oncogenic roles. Consistently, elevated expression of either gene was associated with poorer DFS (Fig 6A). To functionally validate these observations, we knocked down *DEGS1* and *PLOD1* in two LUAD cell lines (Figs 6B and 6C). Silencing either gene markedly suppressed cellular proliferation and migration capacity (Figs 6D-6F). Collectively, these in vitro results indicate that *DEGS1* and *PLOD1* exert oncogenic functions in LUAD, and their inhibition attenuates tumor cell proliferation and migration.

## DISCUSSION

The current management of LUAD relies on tumor-node-metastasis (TNM) stage system, which captures only part of the disease's biologic heterogeneity and explains less than two thirds of prognostic variance.^28^ This limitation underscores the need for biomarkers that reflect tumor biology to refine risk stratification and guide therapy. To this end, we developed MAPI, a metabolic prognostic index that robustly stratified patients with stage I LUAD into distinct groups with divergent survival, molecular features, and therapeutic liabilities.

Metabolic reprogramming is intimately linked to tumor growth, metastasis, and prognosis in LUAD.^29^ We found that Modules 13, 15, and 16 correlated with stage, relapse, and death, suggesting their broad involvement in LUAD progression. Module 13 was enriched for cellular respiration, meeting the enhanced bioenergetic demands of proliferating cancer cells. Notably, oxidative phosphorylation is implicated in multiple metastatic processes.^30,31^ Module 15 was enriched for serine metabolism, which participated in nucleotide biosynthesis, redox homeostasis maintenance, and shaping of immunosuppressive microenvironment, thereby promoting tumor progression.^32^ Module 16 was associated with phospholipid metabolism. Abnormal accumulation of sphingomyelin, one type of phospholipids, decreased membrane fluidity, thereby disrupting contact inhibition and promoting uncontrolled proliferation. Besides, hydrogen barrier formed between sphingomyelin and water molecules may facilitate the survival of circulating tumor cells, eventually promoting metastasis.^33^ Collectively, these modules highlight various mechanisms underpinning LUAD progression.

Most patients with stage I LUAD do not receive adjuvant chemotherapy because TNM stage alone inadequately identifies those at sufficiently high risk to justify toxicity.^34^ Our study sought to address this gap by molecularly defining risk. We found that high-risk patients may derive limited benefit from immunotherapy, but showed increased susceptibility to paclitaxel, bortezomib, and vincristine. A key challenge in cancer treatment is precisely identifying patients who can benefit from specific agents. Bortezomib, a US Food and Drug Administration–approved proteasome inhibitor for multiple myeloma, has shown controversial efficacy in non–small cell lung cancer (NSCLC).^35–38^ Recent studies indicated that its antitumor effect may depend on specific molecular contexts, such as LUAD with elevated MAP17 expression or high NF-κB activity,^39,40^ suggesting potential therapeutic selectivity. Similarly, paclitaxel, the standard first-line agent for advanced NSCLC, has exhibited limited benefit in early-stage trial,^41^ likely because inclusion criteria for the trial relied solely on TNM stage without a detailed distinction at molecular level. Our findings imply that MAPI could identify patients with stage I LUAD likely to benefit from these agents. It is critical to emphasize, however, that these predictions are in silico analyses and remain hypothesis-generating, necessitating further functional and clinical validation.

Our study identified *DEGS1* and *PLOD1* as core MAPI components and validated their protumorigenic functions in vitro. The ablation of DEGS1, a key enzyme in the sphingolipid pathway that converts dihydroceramide to ceramide,^42^ has been shown to induce G0/G1 cell cycle arrest^43^ and promote apoptosis in cancer cells.^44^ Furthermore, its inhibition contributed to the antitumor activity of novel agents such as ABTL0812.^45^ PLOD1 functions as a critical contributor to collagen deposition and cross-link stabilization by hydroxylating lysine residues.^46^ Emerging evidence implicates PLOD1 in tumor progression across multiple malignancies, including hepatocellular carcinoma and gastric cancer.^47–49^ For instance, PLOD1 could drive the aggressiveness of hepatocellular carcinoma through an NF-κB/IL-6/STAT3-dependent mechanism.^47^ Our results thus underscore the key vulnerability in stage I LUAD.

Our study has several limitations. First, the included cohorts were retrospective, and sample sizes for specific biologic validations (eg, scRNA-seq) were limited, so the clinical utility of MAPI warrants prospective validations. Second, biologic mechanisms through which model genes contribute to early-stage LUAD malignancy remain to be elucidated. Third, drug sensitivity analysis was performed in silico and the identified agents were hypothesis-generating, requiring clinical validation. Finally, hyperparameter tuning methods such as nested cross-validation were not performed. Future studies should incorporate such approaches to further optimize model complexity and generalizability.

In conclusion, our study established a novel prognostic model for patients with stage I LUAD, which explains their clinical heterogeneity, facilitates the identification of high-risk patients, and supports the implementation of precision treatment.

## Data Availability

A data sharing statement provided by the authors is available with this article at DOI https://doi.org/10.1200/PO-25-00897. This work analyzed publicly available datasets from TCGA-LUAD, GEO databases (GSE30219, GSE31210, GSE100797, and GSE189357), and EGA database (EGAS00001004006). The full code used in the current study is available at https://github.com/Liu-pc921/early-stage-LUAD-MAPI.git.
